# Antimicrobial resistance in ovine bacteria: A sheep in wolf’s clothing?

**DOI:** 10.1371/journal.pone.0238708

**Published:** 2020-09-03

**Authors:** Nuno Silva, Clare J. Phythian, Carol Currie, Riccardo Tassi, Keith T. Ballingall, Giada Magro, Tom N. McNeilly, Ruth N. Zadoks

**Affiliations:** 1 Moredun Research Institute, Pentlands Science Park, Penicuik, United Kingdom; 2 Institute for Production Animal Clinical Science, Faculty of Veterinary Medicine, Norwegian University of Life Sciences, Sandnes, Norway; 3 Institute of Biodiversity, Animal Health and Comparative Medicine, University of Glasgow, Glasgow, United Kingdom; 4 Sydney School of Veterinary Science, Faculty of Science, University of Sydney, Sydney, Australia; Michigan State University, UNITED STATES

## Abstract

**Background:**

To monitor the prevalence of antimicrobial resistance (AMR), methods for interpretation of susceptibility phenotypes of bacteria are needed. Reference limits to declare resistance are generally based on or dominated by data from human bacterial isolates and may not reflect clinical relevance or wild type (WT) populations in livestock or other hosts.

**Methods:**

We compared the observed prevalence of AMR using standard and bespoke interpretations based on clinical breakpoints or epidemiological cut-offs (ECOFF) using gram positive (*Staphylococcus aureus*) and gram negative (*Escherichia coli*) bacteria from sheep as exemplars. Isolates were obtained from a cross-sectional study in three lowland sheep flocks in Scotland, and from a longitudinal study in one flock in Norway. *S*. *aureus* (n = 101) was predominantly isolated from milk or mammary glands whilst *E*. *coli* (n = 103) was mostly isolated from faecal samples. Disc diffusion testing was used to determine inhibition zone diameters, which were interpreted using either clinical breakpoints or ECOFF, which distinguish the bacterial wild type population from bacteria with acquired or mutational resistance to the compound of interest (non-wild type). Standard ECOFF values were considered as well as sheep-specific values calculated from the data using Normalized Resistance Interpretation (NRI) methodology.

**Results:**

The prevalence of AMR as measured based on clinical breakpoints was low, e.g. 4.0% for penicillin resistance in *S*. *aureus*. Estimation of AMR prevalence based on standard ECOFFs was hampered by lack of relevant reference values. In addition, standard ECOFFS, which are predominantly based on human data, bisected the normal distribution of inhibition zone diameters for several compounds in our analysis of sheep isolates. This contravenes recommendations for ECOFF setting based on NRI methodology and may lead to high apparent AMR prevalence. Using bespoke ECOFF values based on NRI, *S*. *aureus* showed non-wild type for less than 4% of isolates across 13 compounds, and ca. 13% non-wild type for amoxicillin and ampicillin, while *E*. *coli* showed non-wild type for less than 3% of isolates across 12 compounds, and ca. 13% non-wild type for tetracyclines and sulfamethoxazole-trimethoprim.

**Conclusion:**

The apparent prevalence of AMR in bacteria isolated from sheep is highly dependent on interpretation criteria. The sheep industry may want to establish bespoke cut-off values for AMR monitoring to avoid the use of cut-offs developed for other host species. The latter could lead to high apparent prevalence of resistance, including to critically important antimicrobial classes such as 4^th^ generation cephalosporins and carbapenems, suggesting an AMR problem that may not actually exist.

## Introduction

The World Health Organization (WHO) recognizes antimicrobial resistance (AMR) as a global problem in public and animal health [1]. A review on AMR conducted in the UK and often referred to as “the O’Neill Report” based on its lead author [2] estimated that infections caused by resistant bacteria may become the leading cause of death globally by 2050 and estimated the societal and economic cost of AMR at 100 trillion USD in that period of time. To help address the problem, WHO has released a Global Action Plan on AMR with an overall aim of ensuring, for as long as possible, appropriate management of infectious diseases with effective and safe medicines [3], and a call to its member states to develop and implement National Action Plans. In the action plans, the need for a One Health approach is recognized, whereby antimicrobial use in humans and animals are considered jointly, as well as AMR in organisms derived from human and animal hosts and their environments.

To monitor the scale of the AMR problem across countries and species over time, including the potential impact of measures to prevent and reduce AMR, tools are needed to measure the extent and distribution of AMR. Such tools include major reporting infrastructure projects, such as the European Antimicrobial Resistance Surveillance Network (EARS-Net), which is the largest publicly funded system for AMR surveillance in Europe. EARS-Net focuses on data obtained from local and clinical laboratories in European member states and includes AMR data for microorganisms of major public health importance, i.e. *Escherichia coli*, *Klebsiella pneumoniae*, *Pseudomonas aeruginosa*, *Acinetobacter* spp., *Streptococcus pneumoniae*, *Enterococcus* spp., and *Staphylococcus aureus*. In addition, there are national and international networks for reporting of veterinary AMR data, such as the Veterinary Antimicrobial Resistance and Sales Surveillance (VARSS) in the UK, or integrated programs, such as the Danish Programme for surveillance of antimicrobial consumption and resistance in bacteria from animals, food and humans (DANMAP) [4].

Although genetic and genomic methods for AMR monitoring are undergoing rapid development [5], most resistance screening is still based on phenotypic methods. A critical component and potential weakness of phenotypic methods is the need for interpretation of measurements based on inhibitory concentrations or inhibition zones in terms of antimicrobial susceptibility or resistance. One set of interpretation criteria consists of clinical breakpoints as provided by the European Committee on Antimicrobial Susceptibility Testing (EUCAST) or the Clinical & Laboratory Standards Institute (CLSI) for use in human medicine. Those breakpoints have limited clinical relevance in veterinary medicine because of the differences in physiology, pharmacokinetics and pharmacodynamics between host species. Only a few exceptions exist in the form of breakpoints that were developed specifically for use in veterinary medicine, such for ceftiofur use for treatment of bovine mastitis caused by *E*. *coli* or *S*. *aureus* [6]. As an alternative to clinical breakpoints, which are the preferred criteria in clinical microbiology, epidemiological cut-offs (ECOFFS) can be established, in which Normalized Resistance Interpretation (NRI) of minimum inhibitory concentrations or inhibition zone diameters (IZD) is used to identify thresholds to distinguish wild-type (WT) from non-wild type (non-WT) populations [7, 8]. For clarity, use of the terms “susceptible” and “resistant” is reserved for interpretation based on clinical breakpoints throughout this text, whereas the equivalent terms “WT” and “non-WT” are used for interpretation of ECOFF data, which increasingly used in epidemiological studies. The core idea of ECOFFs is that the normal distribution of MIC or IZD values can be used to determine where to draw the line between WT and non-WT isolates. EUCAST guidelines based on this methodology exist and tend to split data from human isolates into clear bi- or multimodal distributions. However, EUCAST recommended cut-offs may cut through the normal distribution of WT data for animal isolates, as demonstrated for *E*. *coli* from wild ungulates [9]. Thus, a proportion of the WT isolates from animals will be classified as non-WT. The high apparent prevalence of resistance for *E*. *coli* from sheep reported in the UK [10], could potentially be an artefact of the use of such ECOFFs.

To explore the hypothesis that cut-off values derived predominantly from human bacterial isolates may result in overestimation of AMR in animal-derived isolates, we assembled collections of *S*. *aureus* and *E*. *coli* as exemplars of gram positive and gram negative bacterial species of major importance to public health. Both bacterial species are also are commonly found in sheep, whereby *S*. *aureus* is a common mastitis pathogen in animals with clinical disease [11] whilst *E*. *coli* is primarily found as gut commensal in healthy animals. Susceptibility data were collected using disc diffusion testing and interpreted based on three criteria, i.e. clinical breakpoints, EUCAST reference values for ECOFF, and sheep-specific ECOFF values calculated from the data using NRI methodology. The estimated prevalence of AMR was compared between methods and suggests significant implications for AMR surveillance in livestock species, whereby current standard ECOFF methods may overestimate the AMR problem in animal agriculture.

## Material and methods

### Sample collection

Samples were collected in Scotland and Norway to obtain sufficiently high numbers of both bacterial target species. In Scotland, two commercial flocks (flock 1 and 2) and the Moredun Research Institute (MRI) research flock (flock 3), mostly comprised of commercial crossbreeds, were selected based on size (at least 50 lactating ewes available for sampling), location (Lowland farms within 1.5 hrs drive from MRI) and the owner’s willingness to participate. Scottish flocks were sampled in cross-sectional studies conducted in 2016, in which faecal samples were collected directly from the rectum from 243 ewes with lambs at foot (54 and 86 ewes from flocks 1 and 2, respectively, and 103 ewes from the research flock). At the same time, milk samples were collected from each mammary gland individually after teat ends were cleaned and disinfected with cotton wool swabs drenched in 70% ethanol. After collection in sterile containers, all samples were maintained in a mobile cooling box before being processed on the same day in the laboratory. The study design was approved by the Moredun Animal Welfare and Ethical Review Body.

On the Norwegian farm, samples were collected during 2014 to 2017 as part of longitudinal monitoring of the health of a research flock of 180 Norwegian White Sheep. Milk samples were collected aseptically from clinical mastitis cases in the first week post-lambing, during the period of peak lactation (approximately 4 to 6 weeks post-lambing) and at weaning time (approximately 5 to 6 months post-lambing). Samples of pus, tissue fluid and vaginal discharges from clinical cases (i.e. diseased animals) arising in the flock were collected directly from affected organs using a sterile swab or by direct aspiration (e.g. arthrocentesis) from both lambs (under 1 year old) and adult sheep (rams and ewes over 1 year old). Udders from ewes that had been recorded and treated for mastitis, or those identified with lumpy udders and mammary abscesses at post-weaning checks were salvaged from the slaughterhouse for standardised bacteriological sampling, performed at NMBU Sandnes within two hours of slaughter. For animals that died on-arm, routine necropsy and standardised bacteriological sampling of the heart, lung, brainstem, spleen, liver and kidneys were performed. Non-invasive milk sampling on the research flock performed by the named veterinary surgeon was permitted under Norwegian Animal Welfare (2015) legislation and approved by NMBU Sandnes ethical committee on use of animals in education and research (29/02/2016).

### Bacterial culture and identification

Milk samples collected in Scotland were lightly vortexed and 10 μL was inoculated onto 5% sheep blood agar (SBA) and MacConkey agar No.3 (Acumedia, Neogen, UK) plates using calibrated sterile plastic loops. Plates were incubated aerobically for 24 to 48 hours at 37 °C. Samples yielding more than 2 phenotypes of bacteria on primary culture were considered contaminated. For culture-positive non-contaminated samples, individual colonies were streaked onto 5% SBA and incubated as above. For faeces samples, approximately one gram was homogenized in 3 mL of sterile saline solution (0.85%) and 10 μL of the suspension cultured on MacConkey agar No.3 plates. Plates were incubated aerobically for 24 to 48 hours at 37 °C. One lactose fermenting colony with *E*. *coli* phenotype was picked from each culture- positive MacConkey plate, and subcultured as described for milk isolates.

Samples from the Norwegian research flock were transported directly to the on-site laboratory for immediate processing by standard bacteriology methods, using initial inoculation and aerobic incubation on blood agar, followed by Gram-staining and limited biochemical testing such as indole, coagulase and oxidase testing. [12]. *S*. *aureus* isolates were obtained from 68 milk (live animal) or udder (post-mortem) samples, and 16 other samples including 15 clinical samples (6 claw abscesses, 2 joint fluid samples, 4 skin wounds/abscesses, and 3 tail abscesses) and one post-mortem lung sample. *E*.*coli* isolates were obtained from 11 milk or udder samples (clinical mastitis cases), two clinical samples (urine and caesarean skin wound), and one post-mortem liver sample (S1 Table). Isolates were frozen at NMBU at -70 °C and later sent by express, chilled transport to MRI for further processing.

At MRI, molecular confirmation of bacterial species identity was conducted on all isolates, regardless of origin, using species-specific primers for amplification of the *nuc* gene, which encodes the thermostable nuclease of *S*. *aureus* [13], or the *uid*A gene encoding the β-glucuronidase enzyme specific for *E*. *coli* and *Shigella* spp. [14]. For DNA-extraction, suspected *S*. *aureus* isolates were plated onto Brain Heart Infusion agar (BD Bacto^™^, Le Pont de Claix, France) and incubated aerobically for 24 to 48 hours at 37 °C. Five to ten individual colonies were re-suspended in 50 μL sterile water containing 100 μg/mL of lysostaphin (Sigma-Aldrich, UK) and incubated at 37 °C for 10 min. Next, 200 μL of 100 mM Tris-HCl, pH 8.0, containing proteinase K (Sigma-Aldrich, UK) was added for a final concentration of 0.05 mg/mL, followed by incubation at 50 °C for 10 min. Enzymes were inactivated by incubation at 95 °C for 5 min. Lysates were briefly centrifuged at 12,000 × g for 3 min and supernatants stored at -20 °C. For suspected *E*. *coli* isolates, genomic DNA was extracted in the same manner but without the lysostaphin step. PCR amplification of *nuc* (for suspected *S*. *aureus*) and *uid*A (for suspected *E*. *coli*) was performed in a final volume of 12.5 μL containing GoTaq Green (Promega, Southampton, UK) and 0.5 μM of each primer using *S*. *aureus* ATCC 29213 and the *E*. *coli* ATCC 25922 as positive controls. The amplicons were examined on 1.2% agarose gel stained with GelRed (Biotium, Fremont, USA).

### Antimicrobial susceptibility testing

One hundred and one *S*. *aureus* and 103 *E*. *coli* isolates (S1 Table) were subjected to antimicrobial susceptibility testing (AST), using the Kirby-Bauer disk diffusion method according to EUCAST guidelines (2018). Briefly, individual colonies of each isolate were collected and suspended in 3 mL of saline solution to a turbidity of 0.5 McFarland and inoculated onto Mueller-Hinton agar (EO LABS, Burnhouse, UK). Antimicrobial agents were added, impregnated in paper disks (Abtek Biologicals Limited, UK), and plates were incubated aerobically at 35 ± 2 °C for 18 to 20 h before measuring IZD with a ruler.

For *S*. *aureus*, the panel consisted of benzylpenicillin (1 U), cloxacillin (5 μg), ampicillin (10 μg), amoxicillin-clavulanic acid (20–10 μg), oxacillin (1 μg), ceftiofur (30 μg), cefquinome (30 μg), cefoxitin (30 μg), erythromycin (15 μg), tilmicosin (15 μg), kanamycin (30 μg), oxytetracycline (30 μg), tetracycline (30 μg), sulfamethoxazole-trimethoprim (23.75–1.25 μg), and enrofloxacin (5 μg). For *E*. *coli*, the panel consisted of ampicillin (10 μg), amoxicillin-clavulanic acid (20–10 μg), aztreonam (30 μg), ceftiofur (30 μg), cefquinome (30 μg), cefotaxime (5 μg), ceftazidime (10 μg), cefepime (30 μg), cefoxitin (30 μg), kanamycin (30 μg), oxytetracycline (30 μg), tetracycline (30 μg), sulfamethoxazole-trimethoprim (23.75–1.25 μg), enrofloxacin (5 μg), and imipenem (10 μg). In addition, *E*. *coli* isolates were tested for extended-spectrum beta-lactamase (ESBL) phenotype using a double-disk diffusion test, which is based on the synergy between third-generation cephalosporins and clavulanate [15]. The reference strains *S*. *aureus* ATCC 29213 and the *E*. *coli* ATCC 25922 were used for quality control.

### Data analysis

To differentiate between susceptible or WT and resistant or non-WT isolates, IZD were interpreted using three sets of criteria, i.e. clinical breakpoints, standard ECOFFs from EUCAST and bespoke ECOFFs calculated from the data using NRI methodology. Clinical breakpoints were taken from EUCAST guidelines when available [16], and from CLSI guidelines if EUCAST guidelines did not exist, i.e. for *S*. *aureus* in combination with amoxicillin-clavulanic acid, ampicillin, cloxacillin, oxacillin, ceftiofur, tilmicosin and enrofloxacin and, for *E*. *coli* in combination with ceftiofur, cefquinome, kanamycin, tetracycline, and enrofloxacin) [6, 15, 17] (S2 and S3 Tables). The breakpoint interpretation for tetracycline was also apply to oxytetracycline. For amoxicillin-clavulanic acid, both EUCAST and CLSI clinical breakpoints were considered because the apparent prevalence of resistance may differ considerably between the two. Likewise, differences have been described for ECOFF values recommended by EUCAST, which are largely based on observations from human clinical isolates, and cut-off values that have been calculated using only data from animals, notably ruminants [9]. Therefore, we used ECOFFs provided by EUCAST [18], as well as sheep-specific cut-off values that were calculated from the data using the NRI method. We refer to the sheep-specific values as cut-off values for wild type (CO_WT_) in the remainder of text, to differentiate them from the ECOFFs recommended by EUCAST. Calculation of cut-off values for IZD using the NRI method is based on the normal distribution of IZDs, which allows for calculation of the mean and standard deviation. We applied the NRI method to the IZDs that were measured for each combination of bacterial species and antimicrobial compound using the guidance and spreadsheet provided for that purpose, and with permission from the patent holder, Bioscand AB, Täby, Sweden (European patent No. 1,383,913, US Patent No. 7,465,559). The CO_WT_ was calculated as the mean IZD for the calculated normalised distributions of a compound/species combination minus 2.5 times its standard deviation, as per standard recommendations [8, 19].

## Results

### Prevalence of resistance based on clinical breakpoints

The reference strains *S*. *aureus* ATCC^®^ 29213 and the *E*. *coli* ATCC^®^ 25922, which were included in all susceptibility tests, showed IZD values within the acceptable range for all antimicrobial agents with available EUCAST criteria. Almost all *S*. *aureus* isolates (96.0 to 100%) were susceptible to the compounds tested based on EUCAST clinical breakpoints (Table 1). Of the *E*. *coli* isolates tested, 80.6% to 100% were susceptible to the compounds evaluated. Antimicrobial susceptibility in less than 90% of isolates was observed for ampicillin, oxytetracycline, and tetracycline (Table 1). If multi-drug resistance is defined as resistance to compounds from at least three antimicrobial classes [20], two (1.9%) of 103 *E*. *coli* had an MDR phenotype, both from Scotland. One of the isolates showed resistance to ampicillin, (oxy)tetracycline, and kanamycin, while the second was resistant to ampicillin, (oxy)tetracycline, and sulfamethoxazole-trimethoprim. ESBL-producing *E*. *coli* or MDR *S*. *aureus* were not detected.

**Table 1:** Prevalence of antimicrobial susceptibility (S) among *Staphylococcus aureus* and *Escherichia coli* of ovine origin based on clinical breakpoints, epidemiological cut-off values (ECOFF) or normalised resistance interpretation (NRI).

Antimicrobial	*S*. *aureus*						*E*. *coli*					
	S^1)^ (%)	EUCAST		NRI			S (%)	EUCAST		NRI		
		ECOFF^2)^	WT^3)^ (%)	CO_WT_^4)^	SD^5)^	WT^6)^ (%)		ECOFF	WT (%)	CO_WT_	SD	WT (%)
Benzylpenicillin	96.0	26	96.0	25	1.7	97.0	-	-	-	-	-	-
Amoxicillin-clavulanic acid^7)^	100.0	-	-	35	1.4	88.1	100.0	16	100.0	15	1.3	100.0
Ampicillin	98.0	-	-	35	1.5	87.1	80.6	14	80.6	10	2.0	97.1
Cloxacillin	100.0	-	-	24	2.1	99.0	-	-	-	-	-	-
Oxacillin	100.0	-	-	19	2.0	97.0	-	-	-	-	-	-
Aztreonam	-	-	-	-		-	99.0	26	97.1	23	2.6	99.0
Ceftiofur	99.0	-	-	17	2.8	99.0	100.0	-	-	19	1.4	100.0
Cefquinome	99.0	-	-	23	1.3	96.0	100.0	-	-	21	2.1	100.0
Cefotaxime	-	-	-	-	-	-	100.0	21	98.1	20	2.1	98.1
Ceftazidime	-	-	-	-	-	-	99.0	20	99.0	19	1.6	99.0
Cefepime	-	-	-	-	-	-	100.0	28	87.4	26	1.3	98.1
Cefoxitin	100.0	22	100.0	23	1.7	99.0	99.2	17	100.0	20	1.1	98.1
Erythromycin	100.0	21	98.0	21	1.6	98.0	-	-	-	-	-	-
Tilmicosin	100.0	-	-	15	1.9	100.0	-	-	-	-	-	-
Kanamycin	100.0	-	-	14	1.6	100.0	98.1	-	-	15	1.4	97.1
Oxytetracycline	97.0	-	-	19	1.5	97.0	89.3	-	-	15	1.1	87.4
Tetracycline	99.0	22	67.3	19	1.3	99.0	89.3	-	-	14	1.3	87.4
Sulfamethoxazole- trimethoprim	100.0	17	100.0	20	2.1	99.2	97.1	21	86.4	21	1.6	86.4
Enrofloxacin	100.0	-	-	21	1.9	100.0	100.0	-	-	21	2.2	100.0
Imipenem	-	-	-	-	-	-	100.0	24	81.6	21	1.5	100.0

^1)^ Susceptible based on clinical breakpoints.

^2)^Epidemiological cut-offs values provided by the European Committee on Antimicrobial Susceptibility Testing (EUCAST).

^3)^Wild type based on ECOFF value provided by EUCAST.

^4)^Cut-off for wild type (mm) calculated using normalised resistance interpretation (NRI) and inhibition zone diameter data from the current study.

^5)^SD = standard deviation.

^6)^Wild type based on CO_WT_ value.

^7)^Clinical laboratory standards institute (CLSI) clinical breakpoint was used for *E*. *coli* isolates (Dias et al., 2015).

(-) data no available.

### Prevalence of resistance based on epidemiological cut-off values (ECOFF)

**Fig 1:**
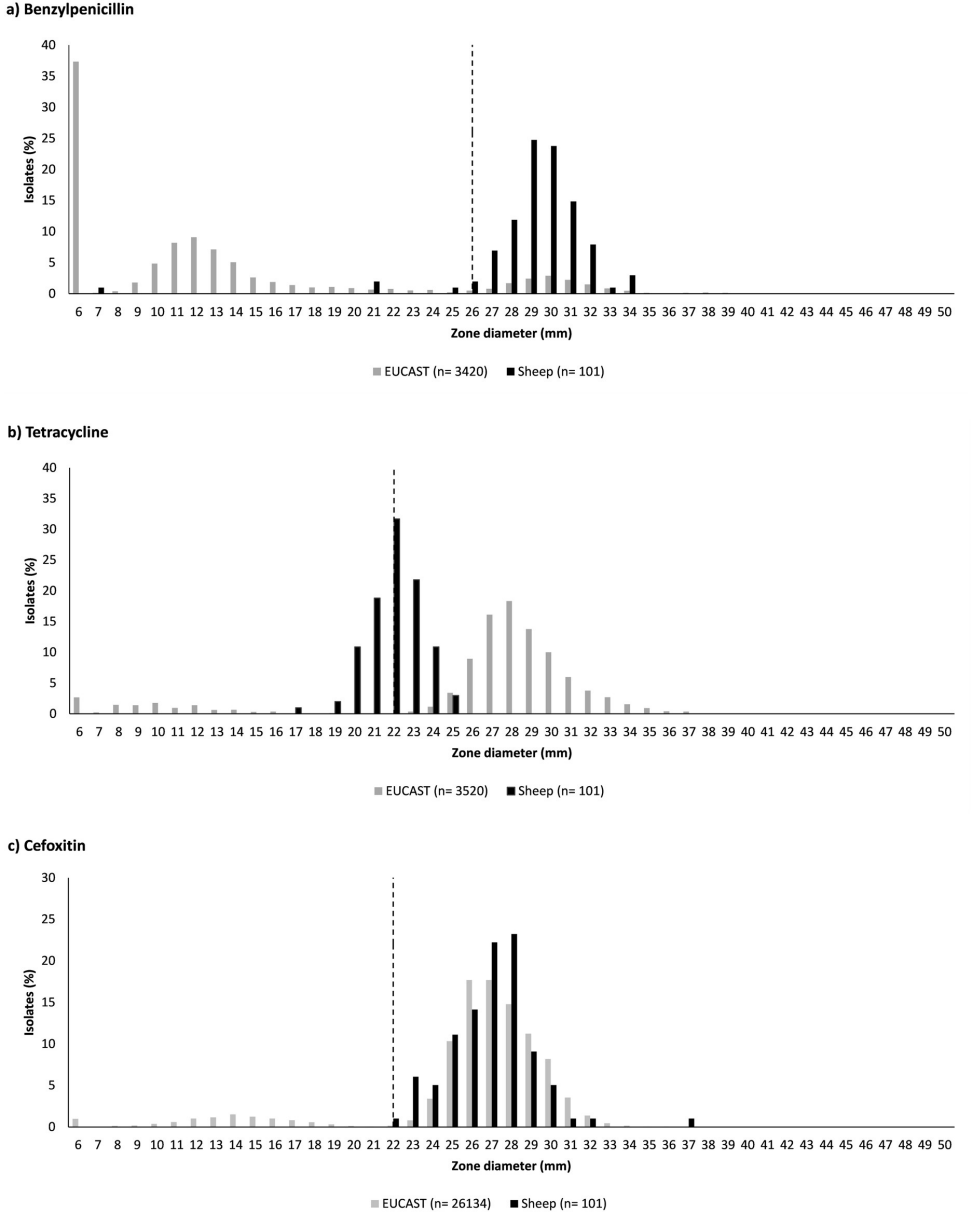
Distribution of inhibition zone diameters for 101 ovine *Staphylococcus aureus* isolates and those available from the European Committee on Antimicrobial Susceptibility Testing (EUCAST). Benzylpenicillin (1 U) (a); tetracycline (30 μg) (b); and cefoxitin (30 μg) (c). Dashed line indicates the epidemiological cut-off values as determined by EUCAST.

**Fig 2:**
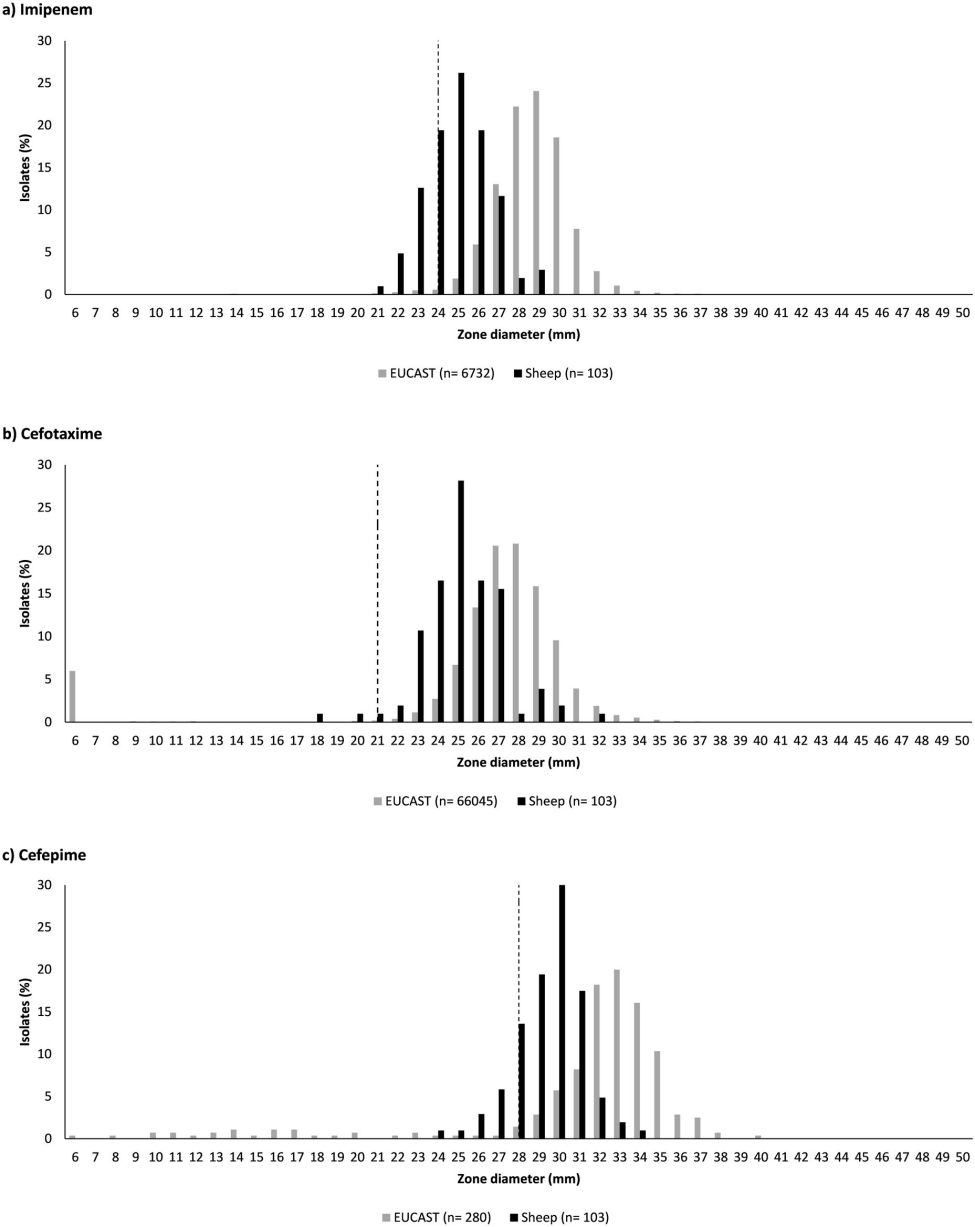
Distribution of inhibition zone diameters for 103 ovine *Escherichia coli* isolates and those available from the European Committee on Antimicrobial Susceptibility Testing (EUCAST). Imipenem (10 μg) (a); cefotaxime (5 μg) (b); and cefepime (30 μg) (c). Dashed line indicates the epidemiological cut-off values as determined by EUCAST.

The distribution of IZDs was unimodal for all compounds in both bacterial species (S2 Table for *S*. *aureus*, S3 Table for *E*. *coli*). Standard deviations from the normalised distributions were well below the recommended threshold of 4 mm [21]. For some compounds, EUCAST-recommended ECOFFs bisected the normal distribution of observed IZD. For *S*. *aureus*, this was the case with IZDs for tetracycline (Fig 1). For *E*. *coli*, this was the case with IZDs for imipenem, which is a carbapenem, and for cefepime, which is a 4^th^ generation cephalosporin (Fig 2).

For *S*. *aureus*, EUCAST provided ECOFF guidelines for 5 of 15 tested compounds. The prevalence of WT phenotype based on ECOFF was high (96.0% to 100%), except for tetracycline, where only 67.3% of sheep isolates were classed as WT (Fig 1, Table 1). NRI-based CO_WT_ values differed from ECOFFs by 0 to 3 mm, whereby NRI-based values could be higher or lower than EUCAST values (Table 1). Based on CO_WT_ values, WT for tetracycline was observed in 99.0% of the isolates, which matches the prevalence of susceptibility based on clinical breakpoint. For other compounds, 96.0% or more of *S*. *aureus* isolates had IZD within the WT range based on CO_WT_, except for amoxicillin-clavulanic acid and ampicillin (Table 1). Prevalence of AMR based on clinical breakpoints was the same as or lower than prevalence of the non-WT phenotype based on ECOFF or CO_WT_, apart from benzylpenicillin, where one isolate was classed as resistant based on the clinical breakpoint but as WT based on ECOFF and CO_WT_. No MDR *S*. *aureus* was identified using ECOFF or CO_WT_.

For *E*. *coli*, EUCAST provided ECOFF guidelines for 9 of 15 tested compounds. The prevalence of WT phenotype was variable and ranged from as low as 80.6% for ampicillin to as high as 100% for amoxicillin-clavulanic acid and cefoxitin (Table 1). With the exception of cefoxitin, NRI-based CO_WT_ values were equal to or lower than EUCAST recommended cut-offs i.e. a narrower inhibition zone was accepted as WT for CO_WT_. As a result, prevalence of the WT phenotype based on CO_WT_ was generally higher than or equal to that based on EUCAST cut-offs (Table 1). Full agreement between the three methods with regards to antimicrobial susceptibility or WT was observed for only two compounds, i.e. amoxicillin-clavulanic acid (using CLSI breakpoint) and ceftazidime (100% and 99.0% susceptible or WT, respectively). For some compounds, e.g. ampicillin, agreement was closer between prevalence based on clinical breakpoints and ECOFF whereas for other compounds, e.g. cefepime and imipenem, agreement was closer for results based on clinical breakpoints and CO_WT_ (Table 1). Based on CO_WT_ values, two *E*. *coli* isolates (1.9%) showed a non-WT phenotype against at least three antimicrobial classes, both from Scotland. Of these two isolates, one was also considered to be MDR based on the clinical breakpoints.

## Discussion

Antimicrobial susceptibility testing of gram positive and gram negative bacteria isolated from healthy and clinically affected sheep using three interpretation criteria showed that AMR in ovine isolates from northern Europe was uncommon. Using clinical breakpoints, only 4.0% of *S*. *aureus* isolates were resistant to benzylpenicillin, which is very low compared to the prevalence of penicillin resistance in international surveillance of *S*. *aureus* from other host species in Europe, including cattle (25% resistance; [22]), dogs (65.2% resistance; [23]) and people (78% resistance in nasal commensal *S*. *aureus*; [24]). Penicillin is among the most widely used antimicrobials in British sheep flocks [25], and remains the drug of first choice for bacterial infections in food-producing animals, including sheep, in Norway [26]. Our data provide no reason to suggest any change to this practice. No resistance to oxacillin and cefoxitin was observed in our isolates. Oxacillin and cefoxitin are surrogate markers for the detection of methicillin-resistant *S*. *aureus* (MRSA) [27, 28] so our results imply that sheep are not an important host species for MRSA. Based on data from European Surveillance of Veterinary Antimicrobial Consumption (ESVAC), both the UK and Norway are among the 5 (out of 31 countries listed) lowest users of antimicrobials in animal production, as expressed in mg per population corrected unit. For that reason, and because the sampling strategy differed between Scotland and Norway so that a sufficiently large number of isolates could be obtained for each bacterial species, comparison of results between the two country was not deemed meaningful.

For *E*. *coli* isolates recovered from sheep, prevalence of AMR was rare (<0.1%) to low (1 to 10%) for most antimicrobial agents tested. Moderate levels (10 to 20%) of AMR to ampicillin, oxytetracycline, tetracycline and sulfamethoxazole-trimethoprim were observed when considering results based on at least 2 of 3 interpretation criteria. The low levels of AMR were similar to those reported from Norway before [26, 29] but contrast with data from the UK Veterinary Antibiotic Resistance and Sales Surveillance report [10], which identified a moderate to very high prevalence of AMR to different classes of antimicrobial agents (penicillins, aminoglycosides, phenicols, tetracyclines and the combination sulfamethoxazole-trimethoprim) in *E*. *coli* isolates from sheep. The discrepancy in AMR reported here and by VARSS may partly result from criteria for selection of isolates for susceptibility testing. For example, VARSS predominantly uses isolates obtained from lambs instead of adult sheep. Bacterial isolates recovered from young lambs and calves, which are physiologically monogastric animals rather than ruminants, can show higher levels of AMR than those from older animals [30, 31]. This might reflect higher exposure to antimicrobial agents in young animals to prevent bacterial neonatal infections, e.g. infectious arthritis (“joint ill”) [32] or *E*. *coli* infection and enterotoxemia (“watery mouth” or “rattle belly”) [33] in some sheep management systems [34]. Age-dependence of AMR prevalence in *E*. *coli*, however, is not necessarily associated with antimicrobial use and may be related to diet and the gut microbiome [30, 35]. For early detection of AMR, screening of lambs may be the method of choice. To represent the AMR prevalence in the national sheep flock, adult animals should be included. As our results were derived from a limited number of selected farms, which may not be representative of the wider sheep or microbial populations, further cross-sectional and longitudinal studies would be needed to test whether the proposed association between age and AMR prevalence exists in sheep and whether AMR in the gastrointestinal microbiota persists as lambs mature. Differences between clinical and carriage isolates may also need to be considered.

In addition to the selection of animals and isolates for AMR surveillance, the choice of interpretation criteria has a major impact on the apparent prevalence of resistance. In medical microbiology and to inform individual treatment decisions, clinical breakpoints are used routinely. They are threshold concentrations that separate isolates with a high versus low probability of treatment success and are often derived from prospective human clinical studies [36]. Such breakpoints are not immediately relevant to non-human isolates. To inform treatment decisions in animals, ideally clinical breakpoints for animal species/bacteria/drug combinations based on PK/PD data and actual clinical outcomes would be available. As an alternative approach, wild-type breakpoints or ECOFFs can be derived from testing of large numbers of isolates [36]. This is also the preferred approach in population level studies, including studies of non-clinical isolates [8, 9]. For example, the European Food Safety Authority (EFSA) uses ECOFFs for interpretation of AMR data from animals and food, which is why we have adopted this approach for ovine isolates [37]. Semi-subjective determination of ECOFF values is possible with the Kahlmeter “eye-ball” method, which is a visual interpretation of IZD distributions [38]. In our study, we used more rigorous statistical methods based on NRI to define sheep-specific CO_WT_ values [8, 39]. ECOFF data are unavailable for many species-drug combinations, which precluded use of EUCAST guidance for close to half of the combinations in our study, even though common organisms and drugs were considered. Calculation of CO_WT_ values was necessary to interpret the remaining data.

When ECOFF values were available, they were often similar or even identical to CO_WT_ values with some exceptions and important ramifications for apparent AMR prevalence. For *S*. *aureus*, the ECOFF of tetracycline was 3 mm larger than the CO_WT_. Isolates are considered non-WT when their inhibition zone is narrower than the threshold value, so a larger threshold value means that more isolates are classed as non-WT. Indeed, almost a third of ovine *S*. *aureus* isolates were classed as non-WT to tetracycline based on ECOFF whereas all but one were considered WT or susceptible based on the CO_WT_ or clinical breakpoint. For this compound, the choice of interpretation criteria has a major impact on apparent AMR prevalence. For *E*. *coli*, the distribution of sheep-derived IZDs was shifted to the left compared to the EUCAST reported distribution, i.e. distribution zones were narrower. The same phenomenon has been reported for wild deer but not for wild birds [9, 40]. It begs the question whether the ruminant gastrointestinal tract, with its complex fermentation system, activates generic efflux pump mechanisms that may provide the WT population with enhanced ability to withstand the action of antimicrobials in the absence of specific acquired resistance mechanisms. The importance of efflux pumps as a common detoxification mechanism employed by bacteria in the gastrointestinal intestinal tract of ruminants has been demonstrated [41], as has their physiological role during the anaerobic adaptation of facultatively aerobic bacteria [42]. Follow-up through *in vitro* or *in silico* analysis of ruminant isolates may shed light on the mechanism underpinning this hypothesis and the phenotypic observations reported here. The observed shift in IZDs for WT isolates is important because the EUCAST-recommended ECOFFs bisected the normal distribution of IZDs for cefepime and imipenem, rather than demarcating the lower boundary of the normal distribution. Cefepime and imipenem belong to the 4^th^ generation cephalosporins and carabapenems, respectively, which are classed by WHO as highest priority critically important antimicrobials or critically important antimicrobials [43]. Thus, a small change in cut-off values has a major impact on the apparent prevalence of resistance to antimicrobials that are of great importance to human medicine, and hence on the apparent contribution of the sheep sector to the global AMR problem. The apparent AMR problem (“wolf”) becomes a non-problem (“sheep”) when clinical breakpoints or bespoke ECOFFs are used. Our findings suggest that the sheep industry may want to establish bespoke cut-off values for AMR monitoring in bacterial isolates derived from sheep in order to avoid the use of cut-offs from human medicine or non-ruminant species. The latter could lead to misclassification and indicate high apparent AMR rates in ovine bacterial isolates, which may suggest an AMR problem where one does not actually exist.

## Supporting information

S1 Table Origin of bacterial isolates by farm, country and sample type. (DOCX)

S2 Table Distribution (%) of inhibition zone diameters (mm) for 101 ovine *Staphylococcus aureus* isolates. Ecological cut-off (ECOFF) values provided by the European Committee on Antimicrobial Susceptibility Testing (EUCAST) are indicated with a thin vertical line if available. Wild type cut-off (CO_WT_) values based on the data from this study and normalised resistance interpretation are indicated with a thick vertical line. Non-wild type populations based on CO_WT_ are shown in light grey and additional non-wild type populations based on EUCAST ECOFFs are shown in dark grey. Data for zone diameters over 44 mm are not shown (all zero).
(DOCX)

S3 Table Distribution (%) of inhibition zone diameters (mm) for 103 ovine *Escherichia coli* isolates. Ecological cut-off (ECOFF) values provided by the European Committee on Antimicrobial Susceptibility Testing (EUCAST) are indicated with a thin vertical line if available. Wild type cut-off (CO_WT_) values based on the data from this study and normalised resistance interpretation are indicated with a thick vertical line. Non-wild type populations based on CO_WT_ are shown in light grey and additional non-wild type populations based on EUCAST ECOFFs are shown in dark grey. Data for zone diameters over 44 mm are not shown (all zero).
(DOCX)
