# Supplementary material for: Antimicrobial resistance in ovine bacteria: A sheep in wolf’s clothing?
Source: PLoS One. 2020 Sep 3;15(9):e0238708. doi: 10.1371/journal.pone.0238708 (PMC7470381; doi:10.1371/journal.pone.0238708)
Supplement: S1 Table — (DOCX) [file pone.0238708.s001.docx]

**S1 Table.** Origin of bacterial isolates by flock, country and sample type

| ***Staphylococcus aureus*** | | | | | | | |  | ***Escherichia coli*** | | | | | |
| --- | --- | --- | --- | --- | --- | --- | --- | --- | --- | --- | --- | --- | --- | --- |
|  | Milk | Udder | Claw abscess | Joint fluid | Skin or soft tissue infection | Tail abscess | Lung |  | Milk | Udder | Faeces | Urine | Surgical wound | Liver |
| **Scotland** |  |  |  |  |  |  |  |  |  |  |  |  |  |  |
| Flock 1 | 4^1)^  3^2)^ | - | - | - | - | - | - |  | 4^2)^ | - | 30^1)^ | - | - | - |
| Flock 2 | 1^1)^  2^2)^ | - | - | - | - | - | - |  | 2^1)^ | - | 24^1)^ | - | - | - |
| Flock 3 | 3^1)^ | - | - | - | - | - | - |  | - | - | 29^1)^ | - | - | - |
| **Norway** |  |  |  |  |  |  |  |  |  |  |  |  |  |  |
| Flock 4 | 36^2)^ | 36^3)^ | 6^2)^ | 2^2)^ | 4^2)^ | 3^2)^ | 1^3)^ |  | 8^2)^ | 3^3)^ | - | 1^2)^ | 1^2)^ | 1^3)^ |
| **Total** | 49 | 36 | 6 | 2 | 4 | 3 | 1 |  | 14 | 3 | 83 | 1 | 1 | 1 |

^1)^ Sample collected from healthy animal

^2)^ Sample collected from animal with clinical signs of disease, e.g. mastitis, arthritis or skin and soft tissue infections.

^3)^ Sample collected at post-mortem
